# Chemotherapy for Late-Stage Cancer Patients: Meta-Analysis of Complete Response Rates

**DOI:** 10.12688/f1000research.6760.1

**Published:** 2015-07-13

**Authors:** Martin L. Ashdown, Andrew P. Robinson, Steven L. Yatomi-Clarke, M. Luisa Ashdown, Andrew Allison, Derek Abbott, Svetomir N. Markovic, Brendon J. Coventry

**Affiliations:** 1 Faculty of Medicine, University of Melbourne, Parkville, Victoria, Australia; 2 Department of Mathematics and Statistics, University of Melbourne, Parkville, Victoria, Australia; 3 Berbay Biosciences, Kew East, Victoria, Australia; 4 ImmunAid Pty Ltd, Fitzroy, Victoria, Australia; 5 Centre for Biomedical Engineering (CBME), University of Adelaide, South Australia, Australia; 6 School of Electrical & Electronic Engineering, University of Adelaide, South Australia, Australia; 7 Melanoma Study Group, Mayo Clinic Cancer Cente, Rochester, MN, USA; 8 Department of Surgery & Tumour Immunology Laboratory, University of Adelaide, South Australia, Australia; 9 Breast, Endocrine & Surgical Oncology Unit, Royal Adelaide Hospital, South Australia, Australia

**Keywords:** Advanced Cancer, Metastatic Cancer, Chemotherapy, Complete Response Rates, Meta-analysis

## Abstract

Complete response (CR) rates reported for cytotoxic chemotherapy for late-stage cancer patients are generally low, with few exceptions, regardless of the solid cancer type or drug regimen. We investigated CR rates reported in the literature for clinical trials using chemotherapy alone, across a wide range of tumour types and chemotherapeutic regimens, to determine an overall CR rate for late-stage cancers. A total of 141 reports were located using the PubMed database. A meta-analysis was performed of reported CR from 68 chemotherapy trials (total 2732 patients) using standard agents across late-stage solid cancers—a binomial model with random effects was adopted. Mean CR rates were compared for different cancer types, and for chemotherapeutic agents with different mechanisms of action, using a logistic regression. Our results showed that the CR rates for chemotherapy treatment of late-stage cancer were generally low at 7.4%, regardless of the cancer type or drug regimen used. We found no evidence that CR rates differed between different chemotherapy drug types, but amongst different cancer types small CR differences were evident, although none exceeded a mean CR rate of 11%. This remarkable concordance of CR rates regardless of cancer or therapy type remains currently unexplained, and motivates further investigation.

## Introduction

Despite intense efforts to improve treatment of advanced cancer over many years with numerous cytotoxic agents and dose regimens, some observers have reported that there has been little substantial improvement in treatment outcomes over the last several decades for most cancer types ^1– 5^. Several notable exceptions exist, where more successful clinical remission and even cure rates have been shown using chemotherapeutic approaches, such as for testicular cancer using platinum-based agents, and acute childhood leukaemia using vinca alkaloids ^6– 9^. Unfortunately, the same has not been true for most other advanced solid malignancies that cause mortality in an estimated 160,000 cancer patients *per week* internationally, with over 11,000 cancer deaths per week in the USA ^10^.

In 2006, Kiberstis and Travis ^1^ commented that “An examination of the annual statistical data compiled by the American Cancer Society quickly reveals that the rate of mortality from cancer has changed very little over the past 50 years,” showing little departure from a point made by Bailar ^2^ from a mortality evaluation of the national cancer program between 1950 and 1990, stating “In the end, any claim of major success against cancer must be reconciled with this [increasing U.S. mortality] figure. I do not think such reconciliation is possible and again conclude, as I did seven years ago, that our decades of war against cancer have been a qualified failure.” Again in 1997, Bailar and Gornik commented, “Observed changes in mortality due to cancer primarily reflect changing incidence or early detection. The effect of new treatments for cancer on mortality has been largely disappointing” ^2^.

This lack of progress persists despite efforts to improve fundamental understanding of cancer growth models ^11– 14^, 1.56 million published papers, and around US $200 billion expenditure on cancer research up until 2006 in the US alone, since the National Cancer Act was passed in 1971 ^1^. This suggests a problem might exist with the current paradigm and the assumption that cytotoxic chemotherapies are acting against cancer cells *per se*, rather than by some other mechanism. In 2010, Lawrence Baker, Professor of Internal Medicine and Michigan Medical School and Chairman of the Southwest Oncology Group stated, “I am trying to get people to stop saying how successful the cancer research enterprise is, it is not true” and “Cure is clearly the expectation of society” ^4^. Cure, or long-term survivals, are associated with the relatively rare event of complete response (CR), where all cancer disappears as a result of chemotherapy.

The above statements are significant when considering standard chemotherapy where CR rates, in late-stage disease, are particularly static and therefore disappointing. Using breast cancer as an example, Frasci *et al.* ^15^ recently reported from the Milan NCI experience, a 7% pathological complete response (pCR) rate using neo-adjuvant combined doxorubicin-paclitaxel and 6% CR rate for advanced breast cancer using an anthracycline-based regimen ^16^. In 1581 patients treated between 1973 and 1982 with consecutive first-line standard-dose doxorubicin and alkylating agent combinations, 263 (16.6%) patients achieved a CR and 49 (3.1%) remained disease free for more than 5 years, and 26 patients (1.5%) remained in first CR at 15 years median follow-up ^17^. A recent study of 2100 patients in 42 phase II trials (70 trial arms) using cytotoxic chemotherapy for metastatic melanoma that completed accrual in the years from 1975 to 2006, conducted by the Southwest Oncology Group, Eastern Cooperative Oncology Group, Cancer and Leukemia Group B, North Central Cancer Treatment Group, and the Clinical Trials Group of the National Cancer Institute of Canada, showed no statistically significant change in progression free survival, or in long-term overall survival over this time period ^18^.

Chemo-resistance metabolic pathways have been widely assumed to be the reason for the development of reduced cytotoxicity against many tumor types ^19^, and may be part of the answer. Clonal genetic diversity and clonal outgrowth of less chemo-susceptible tumor clones is another explanation that has been advanced, related to the existence of tumor stem-cells with the capacity to better adapt and grow in response to environmental selection pressure ^20, 21^. These theories have led to the development of increased dosage regimens, high-intensity dosing, more frequent-dosing regimens, high-dose myelo-ablative chemotherapy with cellular re-infusion methods, and the use of multiple agent chemotherapy regimens. All have become popular in standard medical oncology practice, but there remains little evidence that more chemotherapy is better in terms of clinical outcomes—undesirable toxicity to normal tissues is often a significant problem for the patient, causing treatment limitations and considerable cost in economic and human terms ^22^. Moreover, CR rates and overall survival have not appreciably improved for most individual cancer types, and to our knowledge, wider analysis of CR rates across many different cancer types, or chemotherapy drug types, has not been performed.

### Rationale

Few studies have addressed the reported CR rates over time for systemic cytotoxic chemotherapeutic treatment across a broad range of advanced solid tumours in a systematic manner. Systematic meta-analysis of CR rates across the spectrum of solid tumours and cytotoxic drug types appeared to be lacking in the literature.

### Objective

The objective was to investigate CR rates reported in the literature in clinical trials for advanced cancer treatment across a wide range of cancer types and chemotherapeutic regimens used to date, by conducting a meta-analysis to compare the CR rates and to determine an overall CR rate.

## Materials and methods

We performed this meta-analysis in accordance with the guidelines of the Preferred Reporting Items for Systematic Reviews and Meta-analyses (PRISMA) ^23^.

### Eligibility criteria

Studies were considered initially eligible for evaluation if they were published on the recognized PubMed database, in the English language, and accessible in abstract form. Only full text studies with reported valid randomized series of cases of advanced cancer, treated by described methods of chemotherapy administration alone without concomitant surgery or radiotherapy—both of which may confound data interpretation—and clearly reported CR rates were included, published between 2000 and 2006 inclusive. Study follow-up had to be of sufficient length to permit adequate assessment of CR rates.

### Information sources

The available literature was searched using the PubMed database ( http://www.ncbi.nlm.nih.gov/pubmed/), hosted by the National Center for Biotechnology Information (NCBI), U.S. National Library of Medicine. The search date range was 1st January 2000 to 31st December 2006. This time period “snapshot” was chosen commencing in 2000 because of improved standardization of clinical trial response rate reporting after 1999 with the publication of RECIST criteria ^24^ and before the introduction of newer pathway blocking ‘targeted’ agents from 2007 onwards, which progressively were added to chemotherapy agents, and might have confounded the analysis.

### Search

The following search terms “phase 2/3”, “chemotherapy”, “cancer”, “late stage”, and “complete response” were used. To increase the specificity of the query, specific chemotherapy agents (eg. vinblastine, Taxol, cisplatin, 5FU) were included in the search criteria. The search was restricted to clinical trials, reported in English. Using these criteria, 141 candidate trials were identified by abstract. A spreadsheet containing the gathered data is included in the Supplemental material S1.

### Study selection

Three analysts (MLA, SLY-C and BJC) independently examined those studies that were reported in journals that were accessible by the authors. The CR rates had to be recorded in sufficient detail to enable assessment. Trials involving chemotherapy in combination with surgery or radiotherapy were excluded in order to observe clinical responses to chemotherapy alone. Trials were also excluded if their reported response rates in the text were inconsistent with presented data. Disagreements between the analysts about exclusions were resolved by discussion. After exclusions, sixty-eight clinical trials with a total of 2732 assessable patients remained ^25– 92^.

### Data collection process

Data was extracted as already outlined, using a preliminary screen of two analysts identifying the content validity of each study: advanced cancer, numbers of patients >10 and use of a chemotherapeutic agent or agents alone—with no potentially confounding surgery/radiotherapy or other treatments—for suitable follow-up to clearly report any CR rate. Full texts were obtained and a third analyst scrutinized the papers for the details to verify that the abstract CR rates and reported cases were accurately reported. Any paper that could not meet the above criteria or was otherwise unable to be validated was excluded by agreement. Any discrepancy was solved by repeated review, discussion and agreement. Data was collected in a spreadsheet, de-identified and used for statistical analysis.

### Risk of bias in individual studies

To investigate the risk of possible bias in individual clinical trial studies, we relied on standardized reporting methods of clinical trial results as outlined by WHO and RECIST response rate criteria introduced in 2000 ^24^. The assumption being, all trials would experience similar occurrence of overstatement or understatement of efficacy. As a preliminary guide, to assess feasibility and whether our exclusion criteria might represent bias, we carried out an analysis of CR and Partial Response (PR) data from the published abstracts on the full 130 clinical trials initially identified as eligible. This resulted in an average CR rate of 8.3%, which suggested that more detailed analysis was both feasible and likely to not represent appreciable bias.

### Summary measures

The summary measures used in these studies are the mean CR rate separately determined from meta-analysis of (i) included studies across all cancer types, and (ii) across different primary drug group types specified according to the overall mode of action of each drug type used in the relevant study.

### Synthesis of results

Clinical trial data were entered into a spreadsheet—this spreadsheet can be openly accessed in the Supplemental material S1. Fields recorded for each of the 68 trials include: inclusion/exclusion, %CR, %PR, cancer type, drug type, journal citation etc.

The data were analyzed using a random-effects binomial distribution meta-analysis, for which the response variable was the proportion of patients that were reported to achieve a CR. Separate random-effects binomial distribution meta-analyses were also performed by tumor type and by primary drug type.

We also applied a generalized linear model to the observed number of complete responders for each study, assuming that the distribution of the response variable was conditionally binomial, and using the *logit* link function. We tested the effects of tumor type and primary drug type using marginal likelihood ratio tests, that is, each effect was tested with the other included in the model. The analyses were all performed in R version 2.11.1 ( http://www.r-project.org), an open-source statistical environment ^93^.

### Risk of bias across studies

**Figure 1.**
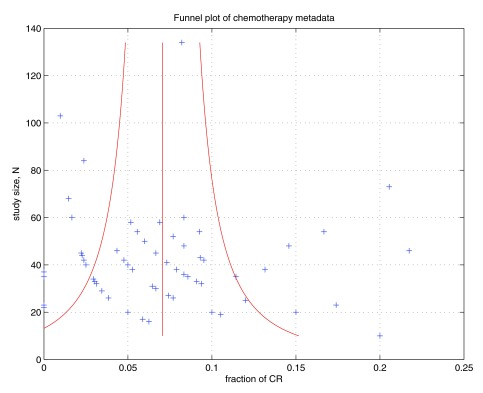
Funnel plot to check for publication bias. For each of the 68 chemotherapy trials, the size *N* of the cohort is plotted on the vertical axis. On the horizontal axis the fraction of the cohort that displayed complete response (CR) is plotted. The red vertical line represents the average of the CR fraction = 0.0706, which corresponds to 7.06%. A standard deviation each side of the average is represented by the curved red lines, calculated by assuming a binomial model. The points appear to be clustered in bands, due to the affect of data quantisation—this is due to the fact that integer numbers of patients have a CR. Thus the first band of points, toward the bottom left of the graph, represent all the trials with a total CR = 1 person, the next band represents all the trials with total CR = 2 people, and so on. For higher total CR numbers, the bands do not appear due to the sparsity of data for large trials. On the funnel plot we see a large spread in CR fraction for 6 trials that are optimistically over a standard deviation, however, the overall skew is nevertheless small. Thus the effects of publication bias appear to be minimal for this study.

To investigate the risk of possible bias by individual clinical trial studies, we undertook a sensitivity analysis. This revealed a surprising consistency of ~7% average CR rate. This suggests, that although the population study with respects to cancer type, age, sex of patient and drugs used, were heterogeneous, the CR rate was fixed or stable at ~7%. To illustrate the possible effects of publication bias we have generated a standard funnel plot ^94, 95^, as shown in Figure 1. Here we see, the cohort size *N* for each chemotherapy trial on the vertical axis and the fraction of the cohort with CR on the horizontal axis. The red vertical line represents the average of the CR fraction = 0.0706, which corresponds to 7.06%. By visual inspection overall skew in the plot is small, and thus the effects of publication bias appear to be minimal for this study.

### Additional analyses

In addition, as a preliminary measure, to see if our exclusion criteria were feasible and whether any bias might exist, we carried out exactly the same analysis on the full 141 clinical trials from CR and PR data in the initial identified published abstracts.

## Results

### Study selection

**Figure 2.**
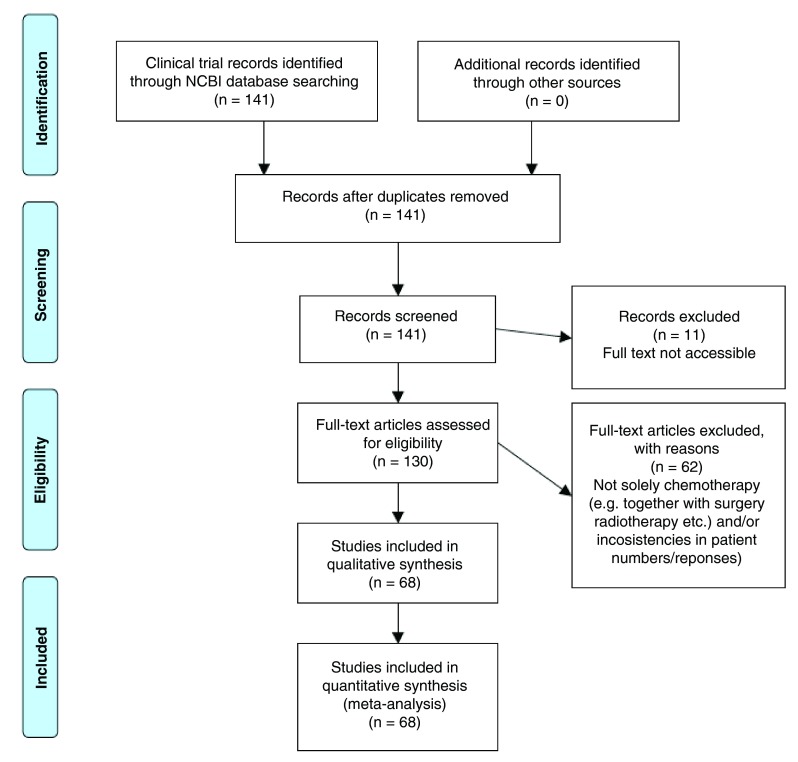
Flow chart summarizing the literature search and study selection for the meta-analysis. The final 68 studies represented a total of 2732 patients.

Referring to the flow chart in Figure 2, we see that from 141 records resulting from the PubMed database search, 11 were not accessible as full text. Of the remaining 130, only 68 of these, without either missing or incomplete data, were evaluable. The 62 exclusions were due to small numbers of patients in the study, treatment of patients by surgery, difficulty substantiating the conclusions from the data provided, or absence of information on precise CR or PR rates. Exclusion were due to: (i) errors or inconsistencies appeared present, (ii) ambiguity in the patient numbers was detected, (iii) unclear staging, (iv) non-advanced cancer was included, or (v) surgery was used—sometimes post-chemotherapy. The final 68 papers were checked for duplicate studies. None were found, and thus no further exclusions were made on this basis.

### Study characteristics

Our analyses involved 68 trials and included studies across more than 10 different cancer types and 7 different cytotoxic drug types. These included by (a) cancer type; squamous cell carcinoma (9 trials), renal cell carcinoma (1) prostate (3), pancreatic carcinoma (6), ovarian carcinoma (11), other carcinoma (9), non-small cell lung carcinoma (4), mesothelioma (1), melanoma (4), gastric carcinoma (6), colorectal carcinoma (6), breast carcinoma (6) and brain malignancies (2); and by (b) primary drug type; topoisomerase inhibitor (TPI), T-Cell cytokine inhibitor (cyclosporine) (TCCi), spindle poison (SP), nucleoside analogue (NA), DNA/RNA synthesis inhibitor (DRsi), antimetabolite (AM), alkylating agent (ALK), antibiotic (AB).

### Synthesis of results

The total number of assessable patients was 2732, of which a total of 193 were classified as complete responders (7.41%). A total of 768 patients were reported as partial responders (28.1%).

**Figure 3.**
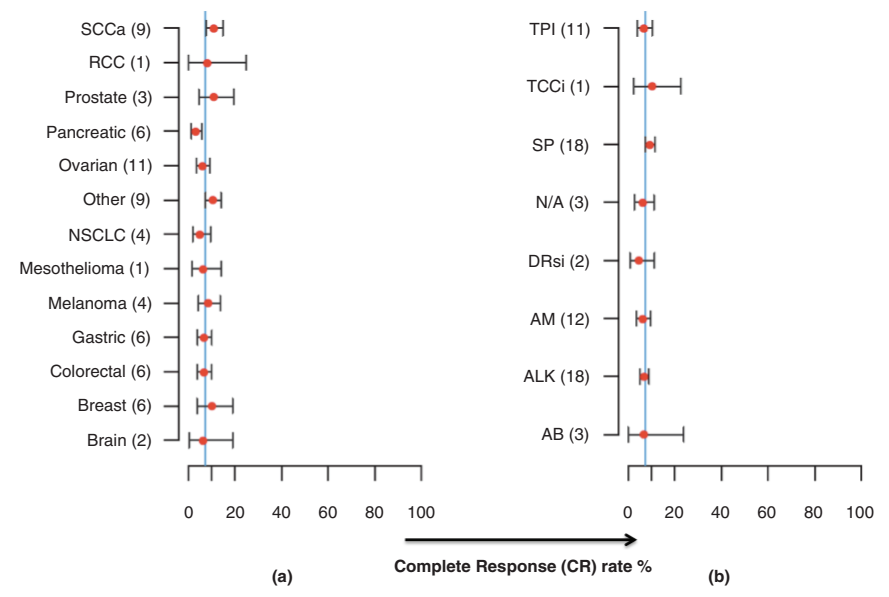
Estimates (red points) and 95% confidence intervals (grey bars) for meta-analyses. These are for ( **a**) Cancer type (SCCa = Squamous cell carcinoma; RCC = renal cell carcinoma; NSCLC = non-small cell lung carcinoma) and by ( **b**) Primary drug type, (abbreviation key; TPI = Topoisomerase inhibitor, TCCi = T-Cell Cytokine inhibitor (Cyclosporine), SP = Spindle poison, NA = Nucleoside analogue, DRsi = DNA/RNA synthesis inhibitor, AM = Antimetabolite, ALK = Alkylating Agent, AB = Antibiotic). The thin vertical blue line denotes a 7.4% CR rate, which is the overall estimate from the meta-analysis.

Individual estimates of mean CR rates within the cancer type groupings were: SCC (10.97%); RCC (8.12%); Prostate (10.9%); Pancreatic (3.2%); Ovarian (6.08%); Other (10.57%); NSCLC (5.02%); Mesothelioma (6.33%); Melanoma (8.48%); Gastric (6.79%); Colorectal (6.71%); Breast (10.14%); Brain (6.45%). These are shown collectively in Figure 3.

The overall meta-analysis estimate of the CR rate from random-effects was 7.41%, [95% confidence intervals (6.27%, 8.64%)].

A generalized linear model that was fitted to the CR rates found a statistically significant relationship between cancer type and CR rate ( *χ* ^2^ = 23.0 on 12 d.f., *p* = 0.028) and no evidence of a statistically significant relationship between drug type/regimen and CR rate ( *χ* ^2^ = 7.87 on 7 d.f., *p* = 0.343).

Despite the statistically significant difference between cancer types, the mean CR rates for each individual cancer type only ranged from 3.2% (pancreatic cancer) to 10.9% (prostate cancer)—see Figure 3.

The ‘other carcinoma’ group comprised cancers of the biliary tract, cervix, uterus, salivary gland, urothelium, and small-cell lung carcinoma. The number of chemotherapy trials using a single agent alone was 17, while the remainder used more than one agent. The number of patients in each trial ranged from 10 to 134, with a median of 37.5.

## Discussion

The complete response (CR) rate has unfortunately remained relatively static for most advanced solid cancers for many decades despite major improvements in understanding considerable new information concerning the molecular genetics, intracellular pathways, adhesion mechanisms, stromal characteristics, angiogenesis, metastatic processes, and immunology, relating to many cancer cell types and modalities of treatment. Similarly, advances in organic chemistry, molecular structural crystallography, synthesis, and pharmaceutical production have not led to expected rapid advances. Numerous different, often quite ingenious, approaches have failed to significantly improve CR rates for survival in patients with advanced cancers ^20^. With this in mind we performed a meta-analysis of existing trials and treatment modalities across most common cancer types over the seven years between 2000 and 2006 inclusive.

### Summary of evidence

In this paper, we report the results of a meta-analysis of 68 chemotherapy trials for cancer treatment, in which we sought to evaluate the CR rate for late-stage cancer patients receiving chemotherapy, embracing a wide variety of solid tumors and drug types. Overall, the CR rate for patients with most types of late-stage cancers receiving chemotherapy are between 5% and 10%. Our meta-analysis suggests that the CR rate for patients with most types of late-stage cancers receiving chemotherapy are between 5% and 10% across many cancer types, with an actual mean CR rate of 7.41% approximately bisecting this estimated range.

This study did not set out to include cancers that represent rare notable exceptions, such as testicular carcinoma and childhood acute lymphoblastic leukemia, which are known to be highly chemo-responsive with CR rates of around 80–90% ^8, 9^. However, most advanced solid tumors in adults (eg. colon, breast, prostate, melanoma, lung) are unfortunately typically lethal with CR rates that are almost reciprocal to those mentioned. The common advanced solid cancers, therefore, formed the focus of the present meta-analysis.

The CR rate was surprisingly concordant across the trials despite wide differences in tumor type, chemotherapy combination and mechanism of drug action. This suggests that the range of agents being used is low in direct anti-tumor activity. That finding has been explained by possible development of ‘chemo-resistance’, however, it appears to occur in about 93% of patients for the majority of tumour types and agents. An alternative explanation is interference with the host immune system in an adverse manner, which would abrogate generation or perhaps diminish the effectiveness of an existing anti-tumor immune response ^96^. Many systemically administered chemotherapeutic agents exert their effects non-specifically on many rapidly dividing tissues other than the cancer itself. Anti-angiogenic activity or direct injury to intra-tumoral blood vessels has been proposed as the mechanism of action of some agents, while others induce DNA or mitochondrial damage.

Unfortunately, selectivity for the malignant cells *in vivo* is often poor and it has been widely appreciated that injury to normal tissues occurs together with tumor cell damage, causing typical side effects such as nausea, diarrhea, marrow suppression, stomatitis, mucositis, and hair loss ^22^. Some of these may be dose limiting and often significantly reduce the quality of life of the patient. In addition to marrow suppressive effects, recent data indicates that the immune system is often injured during chemotherapy treatment, with reduction in the white blood cell count being commonly noted. Leucopaenia may be severe, but is sometimes more subtle, and in particular T-cells can be ablated ^20^. Subsets of rapidly dividing T-cells may be particularly vulnerable to injury, and paradoxically, may be even more susceptible than the cancer cells. If the ablated cells are effector T-cells, then any effective immune response may be eliminated or reduced. However, if regulatory T-cells are selectively ablated or depleted, then the T-effector response may be enhanced ^96– 98^. Global depletion of both groups of T-cells may also result in the reduced ability to mount an effective immune response, and this may possibly ‘re-set’ the immune response.

A total of 768 patients were reported as partial responders, with an estimated partial response rate from the meta-analysis of 27.9%—and when combined with complete responders (CR + PR) providing an overall response rate of 35.3%. Effectively, 64.7% of patients were ‘non-responders’ to therapy by not achieving a measurable clinical response. The heterogeneity of clinical responses might reasonably be explained by the random manipulation of the immune system being determined by mathematical probability when chemotherapeutic regimens are randomly applied without consideration of which particular subsets of susceptible immunological cells are actively proliferating at the time of each dosing ^20, 96– 100^.

In order to be confident that our systematic exclusions did not skew the results unreasonably, we repeated the meta-analysis using the abstracts from the full complement of 130 papers for which suitable CR results were provided within the summaries. The estimated CR rate was 8.43%, with 95% confidence interval (7.18%, 9.78%), which differed only slightly from the rate reported for the studies that were within the scope of the full-article 68 trial meta-analysis.

### Limitations

The following caveats should be considered when interpreting our results. Inevitably, the definition of CR varied slightly amongst some studies. The CR was usually defined as complete regression of all detectable tumour clinically or radiologically, while PR was defined more variably. The studies were not necessarily a random sample because they were selected from the PubMed database between 2000 and 2006 and required full-text availability with adequate data for the purposes of the current studies, introducing the potential for inadvertent selection and publication bias. However, every effort was made to avoid this problem. Finally, the analyses included here were the reported clinical response statistics rather than the reported rate estimates and confidence intervals. Hence the analysis might conflict with those reported in the actual paper. A larger study may overcome/diminish such biases.

Another point is that this study is limited to papers from the PubMed database. The usual recommendation for meta-analyses is to use multiple databases ^101^. However, this is with the intention of mitigating against the omission of negative results that may bias association studies (eg. establishing a positive association between a cancer and a genetic marker). However, this paper is not an association study; we are merely demonstrating the observation that complete response rates for chemotherapy are low across a reasonably broad range of literature. The funnel plot in Figure 1 demonstrates lack of bias as there are roughly equal numbers of points higher and lower than the two standard deviation lines.

## Conclusions

The knowledge gained from this meta-analysis offers a broad view of the effects of chemotherapy on CR rates for cancers of specific types; collectively for many types of cancers; and for chemotherapy agents with different mechanisms of action. It is particularly noteworthy that the confidence intervals lie progressively closer to the respective mean CR rate for the agents that have historically been in use for the longest time and for which the most clinical trial data exists. This indicates that the agents that have been in use the longest time have a ’real’ CR rate that approximates 7%—an interesting result despite years of use of these agents, even in multiple combination regimens. The potential for variability amongst the individual clinical studies included in the meta-analysis is recognized, however, selection of higher quality valid clinical trials using chemotherapy alone as the sole treatment modality aimed to reduce the natural heterogeneity amongst the studies. In this meta-analysis we further sought to include many cancer types to purposely examine CR rates within a spectrum of cancers. The data offer what we contend is a relatively unbiased and ‘clean’ view of representative ‘real world’ clinical data, from a wide range of sources—indicative of true international clinical experience. The funnel plot indicates that this objective satisfies optimal minimisation of publication bias.

The significance of the results of our meta-analysis are that across different tumour types, and regardless of different chemotherapy agents/approaches, the CR rates to cancer have remained essentially static and locked at about 7%, despite over 7 years of diligent clinical effort during which these trials were conducted. This might suggest that probabilistic effects are operating to mathematically restrict the ability to manipulate the clinical CR rate—if this can be further understood or overcome, CR rates may potentially be capable of being significantly increased.

The data might offer an alternative approach to thinking about the possible mechanisms of action of chemotherapy, irrespective of their direct effects on the tumour cells. In considering the consistently low mean CR rates of 7.4% across many tumor types, and using different chemotherapy agents, the findings are suggestive that the paradigm of chemotherapy directly acting for tumour cell killing per se, might be incorrect. Rather, the effects of chemotherapy on immunological cells at any point in time may be of considerable significance, depending on whether rapidly proliferating T-effector cells or T-regulatory cell subgroups are selectively ablated by cytotoxicity, as has been reported in some mouse studies ^99, 100^. The balance of the anti-tumor immune response may be pivotally controlled by the relative ablation of either effector or regulatory cell populations, and thereby determine the growth or destruction of the tumor. Indeed, higher doses of cytotoxics might ablate effector T-cells, thereby blunting the immune response and the ability of the patient’s own immune system to be effective. The recent findings concerning the efficacy of metronomic low-dose chemotherapies, administered more frequently, regularly and chronically, would also suggest that the immune system may be pivotal in determining the anti-tumor effect and clinical outcome of the patient ^102^.

We are currently taking this approach clinically with some success, and are actively investigating the possible timing of chemotherapy and immunotherapy ^103– 105^. Optimally timed doses are capable of manipulating the immune response by selective immuno-stimulation or immuno-ablation. The intention is to maximize the *in-vivo* T-effector response, while minimizing the T-regulatory responses, within the patient, to provide a potential means of synchronizing the ongoing immune response in the patient for improving clinical outcome ^20, 96^.

The main finding of this study is the remarkable concordance of CR rates amongst studies of patients with different cancer types, and also amongst a range of cytotoxic chemotherapy types. This notable similarity in CR rates regardless of cancer or therapy type remains currently unexplained, and requires further intensive investigation.

## Additional information

**Supplementary information** accompanies this paper as follows:

***S1 Spreadsheet of collated information.*** The spreadsheet contains the raw collated information from the 68 papers included in this study. The columns are described as follows: (A) paper/trial number in order of search download, (B) paper citation details, (C) year published, (D) N = number of patients in trial, (E) CR, number of complete responses post therapy reported in trial, (F) PR, partial responses in trial, (G) cancer type, (H)-(L) chemotherapeutic agent/s used in trial, (M) drug type, (N) drug/s used as mono or multi agent combination.

.

***S2 PRISMA checklist.*** Standard checklist for compliance to PRISMA standards for the meta-analysis (available at http://www.prismastatement.org/statement.htm).
