# Supplementary material for: Chemotherapy for Late-Stage Cancer Patients: Meta-Analysis of Complete Response Rates
Source: F1000Res. 2015 Jul 13;4:232. [Version 1] doi: 10.12688/f1000research.6760.1 (PMC4706056; doi:10.12688/f1000research.6760.1)
Supplement: Peer review history

# Peer review: Referee response for version 1

**DOI:** 10.5256/f1000research.7263.r11333

**Published:** 2015-11-25

**Authors:** Gábor Balázsi

**Affiliations:** 1 The Louis and Beatrice Laufer Center for Physical and Quantitative Biology, Stony Brook University, Stony Brook, NY, USA

The statistical analysis is appropriate and rigorous. The results are interesting – similar rates of CR for a variety of cancers and drug types. I would like to recommend indexation after a few minor comments have been addressed:

1. It would be interesting and important to include a statement on the rate of Spontaneous Regression (tumour regressing without any treatment). This could simply be extracted from previous publications (no additional analysis needed). This would answer the question: does chemotherapy provide any benefit compared to no treatment at all? It would be interesting to add a sentence on comparing the rates of CR and SR.
2. In the Introduction, a sentence on the molecular mechanisms (such as gene regulation) leading to metastasis would be useful. A relevant citation could be Lee *et al. *(2014).
3. It would also be interesting to add a sentence on any trends observable over time. If the data are insufficient to make a statement, then the authors should write a sentence stating that.

I have read this submission. I believe that I have an appropriate level of expertise to confirm that it is of an acceptable scientific standard.

## References

[1] LeeJLeeJFarquharKSYunJFrankenbergerCABevilacquaEYeungKKimEJBalázsiGRosnerMR: Network of mutually repressive metastasis regulators can promote cell heterogeneity and metastatic transitions. *Proc Natl Acad Sci U S A*.2014;111(3) : 10.1073/pnas.1304840111 E364-73 10.1073/pnas.1304840111 24395801PMC3903259

# Response

**Published:** 2015-12-01

**Authors:** Derek Abbott

**Affiliations:** University of Adelaide, Australia

The authors respond to Professor Balázsi's suggestions as follows:

1. In clinical terms, spontaneous regression is an exceedingly rare event that an individual clinician might perhaps be lucky enough to observe in an entire career of clinical practice - many do not. As such, it is therefore far less than the CR rates that we have identified and reported here in this meta-analysis, and the CR rates are most likely to be the effect(s) of treatment.
2. We agree that molecular mechanisms and gene regulation are important aspects in neoplasia and oncogenesis, but these aspects are not precisely central to the subject of our meta-analysis, and so we did not stray into those areas in this paper
3. This is an interesting point, however, since we necessarily confined our studies to the time period from 2000 - 2007, we did not see a trend over that short time. However, the suggestion is a particularly interesting one and potentially relevant over a longer period. This is an open question for future study.

# Peer review: Referee response for version 1

**DOI:** 10.5256/f1000research.7263.r9467

**Published:** 2015-08-12

**Authors:** Joseph J. Drabick

**Affiliations:** 1 Department of Medicine, Division of Hematology/Oncology, Penn State Milton S. Hershey Medical Center, Hershey, PA, USA

This is a fascinating review by Ashdown and colleagues that examines the CR rate to various cytotoxic regimens for various cancers as part of meta-analysis focusing on the attainment of CR. The remarkable concordance of CR rate across cancer and therapy types is very intriguing. The authors explore possible explanations for this phenomenon and surmise it is most likely immune in nature. This is a remarkable finding that begs for some solution and to my knowledge is a novel observation.

I have read this submission. I believe that I have an appropriate level of expertise to confirm that it is of an acceptable scientific standard.
